# Immunohistochemical ERG positivity is associated with decreased PSMA expression and lower visibility in corresponding [^68^Ga]Ga-PSMA-11 PET scans of primary prostate cancer

**DOI:** 10.1007/s00259-024-06856-x

**Published:** 2024-07-31

**Authors:** Niels J. Rupp, Sandra N. Freiberger, Daniela A. Ferraro, Riccardo Laudicella, Jakob Heimer, Urs J. Muehlematter, Cédric Poyet, Holger Moch, Daniel Eberli, Jan H. Rüschoff, Irene A. Burger

**Affiliations:** 1 Department of Pathology and Molecular Pathology, University Hospital Zurich, Schmelzbergstrasse 12, 8091 Zürich, Switzerland; 2 University of Zurich, Zurich, Switzerland; 3 Department of Nuclear Medicine, University Hospital Zurich, University of Zurich, Zurich, Switzerland; 4 Department of Radiology and Oncology, Faculdade de Medicina FMUSP, Universidade de Sao Paulo, Sao Paulo, Brazil; 5 Department of Biomedical and Dental Sciences and Morpho-Functional Imaging, Nuclear Medicine Unit, University of Messina, Messina, Italy; 6 Seminar for Statistics, Federal Institute of Technology (ETH) Zurich, Zurich, Switzerland; 7 Department of Urology, University Hospital Zurich, University of Zurich, Zurich, Switzerland; 8 Department of Nuclear Medicine, Cantonal Hospital Baden, affiliated Hospital for Research and Teaching of the Faculty of Medicine of the University of Zurich, Baden, Switzerland

**Keywords:** Transcriptional Regulator ERG, Glutamate carboxypeptidase II, Immunohistochemistry, Neoplasm staging, Positron emission tomography, Prostatic neoplasms

## Abstract

**Purpose:**

*TMPRSS2:ERG* gene fusion negatively regulates PSMA expression in prostate adenocarcinoma (PCa) cell lines. Therefore, immunohistochemical (IHC) ERG expression, a surrogate for an underlying *ERG* rearrangement, and PSMA expression patterns in radical prostatectomy (RPE) specimens of primary PCa, including corresponding PSMA-PET scans were investigated.

**Methods:**

Two cohorts of RPE samples (total *n*=148): In cohort #1 (*n*=62 patients) with available RPE and preoperative [^68^Ga]Ga-PSMA-11 PET, WHO/ISUP grade groups, IHC-ERG (positive vs. negative) and IHC-PSMA expression (% PSMA-negative tumour area, PSMA_%neg_) were correlated with the corresponding SUV_max_. In the second cohort #2 (*n*=86 patients) including RPE only, same histopathological parameters were evaluated.

**Results:**

Cohort #1: PCa with IHC-ERG expression (35.5%) showed significantly lower IHC-PSMA expression and lower SUV_max_ values on the corresponding PET scans. Eight of 9 PCa with negative PSMA-PET scans had IHC-ERG positivity, and confirmed *TMPRSS2::ERG* rearrangement. In IHC-PSMA positive PCa, IHC-ERG positivity was significantly associated with lower SUV_max_ values. In cohort #2, findings of higher IHC-PSMA_%neg_ and IHC-ERG expression was confirmed with only 0-10% PSMA_%neg_ tumour areas in IHC-ERG-negative PCa.

**Conclusion:**

IHC-ERG expression is significantly associated with more heterogeneous and lower IHC-PSMA tissue expression in two independent RPE cohorts. There is a strong association of ERG positivity in RPE tissue with lower [^68^Ga]Ga-PSMA-11 uptake on corresponding PET scans. Results may serve as a base for future biomarker development to enable tumour-tailored, individualized imaging approaches.

## Introduction

Prostate-specific membrane antigen (PSMA-) PET has been shown to be an effective modality for staging and restaging of prostate cancer (PCa). However, in up to 10% of primary PCa, PSMA uptake is absent and therefore cannot be detected by PSMA-PET [1, 2]. As we and others have shown, PSMA expression patterns (≥ 20% PSMA negative tumour area, PSMA_%neg_) are strongly associated with negative PET scans. Other parameters correlated with lower PSMA-PET uptake in RPE specimen of primary PCa were infiltrative growth pattern, smaller tumour size and WHO/ISUP grade group 2 [3–5]. So far, little is known about regulation of PSMA expression, however DNA damages, interaction with androgen receptor signalling and epigenetic changes have been described [6–10]. In addition, *TMPRSS2::ERG* gene fusion has been shown to negatively regulate PSMA expression in cell lines [11]. Recurrent gene fusions between *TMPRSS2* (transmembrane protease serine 2) and the gene *ERG*, a member of the transcription factor erythroblastosis virus E26 transforming sequence family (ETS), are highly specific to PCa and can be found in up to 40-50% of prostate carcinomas [12–14]. Immunohistochemical (IHC) ERG expression is thereby a robust surrogate marker for the detection of *TMPRSS2::ERG* gene fusions [15–17]. Therefore, the aim of the present study was to investigate the association of ERG and PSMA expression in tissues of RPE specimens, and their correlation with the corresponding [^68^Ga]Ga-PSMA-11 PET scans.

## Materials and methods

### Study population (Cohort #1 and #2)

#### Cohort #1

To identify the correlation between immunohistochemical profiles and [^68^Ga]Ga-PSMA-11 PET, we selected all patients that underwent a [^68^Ga]Ga-PSMA-11 PET for newly diagnosed intermediate or high-risk PCa at the University Hospital Zurich from April 2016 to May 2018. Patients were part of a retrospective analysis for morphological patterns and PET quantification and have been published previously (*n*=62) [3]. Patients without a radical prostatectomy (RPE) specimen available were excluded. The study was approved by the local Cantonal Ethics committee (BASEC Nr. 2018–01284, BASEC Nr. 2022-1778). A general written informed consent for the use of their material and data was signed by all included patients. Data collected encompassed operating date, tumour (T-) stage, WHO/ISUP prognostic grade groups / (modified) Gleason score, tumour size (mm^2^) of the PCa on the selected histological slide. Small tumours on histology (< 5 mm) were excluded for correlation with the PET examination.

#### Cohort #2

A second cohort with *n*= 86 consecutive patients from the ProCOC (the prostate cancer outcomes cohort) study [18] was selected (Ref. Nr. StV KEK-ZH-Nr. 06/08) to further investigate the findings from cohort #1, and to avoid the (intermediate- to high-risk) selection bias from cohort #1. All patients signed the study consent for the use of their tissue and data. In brief, selected patients with localized PCa, from whom a radical prostatectomy specimen in the years 2008 - 2011 was available, were included.

### Histopathological parameters and Immunohistochemistry (Cohort #1 and #2)

**Fig. 1:**
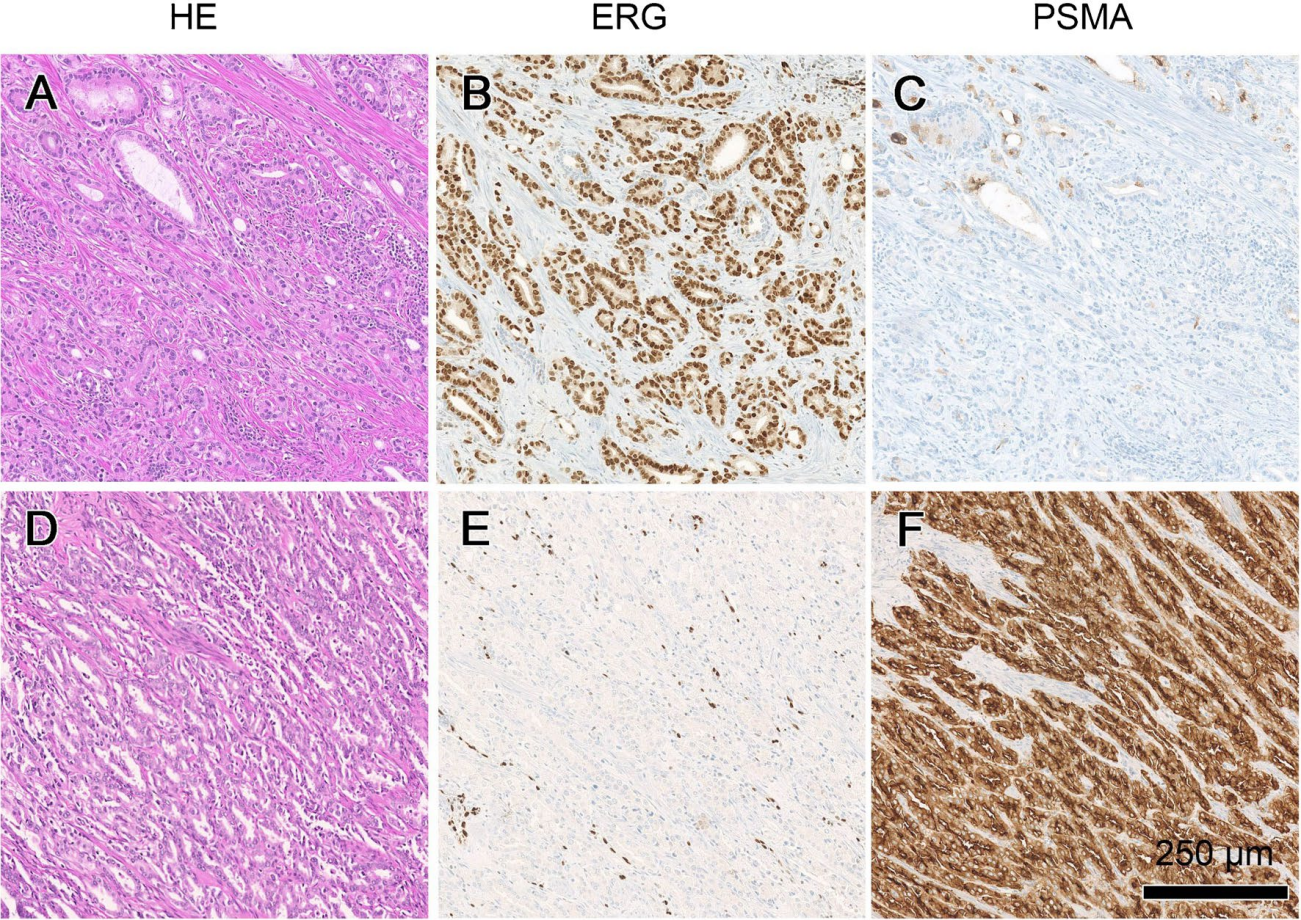
Overview of two prostate adenocarcinoma cases differentially expressing ERG and PSMA. The first case (**A**) shows nuclear ERG reactivity in the tumour cells (**B**), and vastly negative PSMA expression (**C**). The second case (**D**) depicts no ERG expression in the tumour (**E**) with internal endothelial positive control, and diffuse PSMA reactivity (**F**). Scale bar: 250 μm

A total of *n*=148 formalin-fixed, paraffin-embedded (FFPE) RPE specimens (Cohort #1: *n*=62, Cohort #2: *n*=86) were evaluated on 2 μm hematoxylin and eosin (H&E)-stained sections. One representative slide from the RPE specimen with the largest tumour size (mm^2^; dominant tumour lesion) was selected for further investigation. Grading and staging were performed according to the WHO/ISUP/UICC guidelines [19, 20]. The dominant tumour lesion was graded separately and included in the analysis. Immunohistochemical staining for PSMA (DAKO, M3620, clone 3E6, 1:25) was performed as described previously [3]. The predominant PSMA expression patterns were visually quantified using a four-tiered system (0 = negative, 1+  = weak, 2+  = moderate, 3+  = strong) for both membranous and cytoplasmic PSMA expression. Furthermore, tumour areas without PSMA expression were quantified in steps of 5%, 10% and further 10% increments in relation to the total tumour area, as percentage PSMA-negative tumour area (PSMA_%neg_) as a consent of two evaluating pathologists (N.J.R., J.H.R) (Fig. 1). Heterogeneity was defined by differences in the staining pattern of at least 5% of the representative tumour slide. ERG (EPR3864, Roche, prediluted) expression was evaluated in tumour cells using a two-tiered system (positive/negative), whereas endothelial cells and lymphocytes served as an internal positive control (Fig. 1). ERG scoring was categorized in negative and positive, as described previously [16]. Evaluations were done by two board-certified, experienced genito-urinary pathologists (N.J.R., J.H.R). Slides were digitalized (Nanozoomer NDP digital slide scanner C9600-12) using the Hamamatsu NDP.view 2.8.24 Software.

### Sequencing (Cohort #1)

To investigate the association of ERG expression status and molecular *ERG* rearrangement, the subgroup of the PSMA PET negative cases was sequenced using the RNA part of the Oncomine Focus assay (ThermoFisher Scientific, Waltham, MA, USA). In brief, RNA was extracted from FFPE tumour tissue using the automated Maxwell 16 LEV RNA FFPE Purification Kit (Promega, Madison, WI, USA). Library preparation for the Oncomine Focus Asay was performed according to the manufacturer's manual using 10 ng of RNA as input material. The Ion Chef Instrument was used for templating the libraries on Ion 540 chips and sequencing was performed using the Ion S5 system (both ThermoFisher Scientific, Waltham, MA, USA). Sequencing data were analysed using the IonReporter software (ThermoFisher Scientific, Waltham, MA, USA) with the generic filter settings.

### Imaging (Cohort #1)

All patients underwent clinical routine [^68^Ga]Ga-PSMA-11 PET/computed tomography (CT) (Discovery VCT 690 PET/CT (GE Healthcare, Waukesha, WI, USA) or Discovery MI PET/CT (GE Healthcare, Waukesha, WI, USA)) or a [^68^Ga]Ga-PSMA-11 PET/MRI (SIGNA PET/MR, GE Healthcare, Waukesha, WI, USA) after a single injection of [^68^Ga]Ga-PSMA-11 (2 MBq / kg) as described previously [3].

### Imaging analysis (Cohort #1)

Imaging analysis was performed on a dedicated workstation (Advantage Workstation, Version 4.7, GE Healthcare). Exact location of the selected dominant lesion was given by the pathologist (anterior/posterior, left/right, base, midglandular or apex). All lesions were reviewed together with the histopathology slides in a multidisciplinary meeting. A volume of interest (VOI) was placed over the lesion, if clearly visible on PSMA PET. If lesions were not clearly delineated on PET images, the VOI was placed over the tumour region, in correspondence to the lesion localization on histopathology. To quantify [^68^Ga]Ga-PSMA-11 uptake the maximum standardized uptake value (SUV_max_) was measured. There is a wide range of proposed cutoffs to detect significant PCa from SUV_max_ 3.15 [4] to up to SUV_max_ 9.1 [21]. Uptake in the central zone was not included in the study. As in our previous works we decided to set a cutoff for PSMA uptake at SUV_max_ ≥ 5 for the definition of PSMA-PET positivity [3].

### Statistical analysis

Distributions of variables were assessed visually. Due to right-skewness of variables, univariate comparisons of continuous variables between two groups were performed with the Mann-Whitney U test. For the comparison of a continuous variable among multiple groups, a Kruskal-Wallis test with a Bonferroni-Holm adjusted posthoc test was calculated. The Fisher Exact test was used to investigate associations between two binary factors, due to observed cell counts < 5 *P*-values <= 0.05 were considered significant. Correlations were performed using R (4.3.2). Graphs were generated using R (4.3.2).

## Results

### Study population

#### Cohort #1

A total of 62 patients were identified with preoperative [^68^Ga]Ga-PSMA-11 PET and RPE available for immunohistochemical analysis, for flow chart see previous publication [3]. Patients were scanned with a mean dose of 130 ± 18 MBq, range 81–171 MBq. Tumour selection was done as previously described [3]. Clinico-pathological characteristics are shown in Table 1. The selected tumour regions were graded as WHO/ISUP grade groups from 2 to 5 (Table 1). Four lesions were excluded for correlation analysis between histology pattern and [^68^Ga]Ga-PSMA-11-PET uptake, as the size was below 5 mm. SUV_max_ values ranged from 3.1 to 48.4 (mean 14.96 ± 10.8).

**Table 1:** Clinicopathological characteristics of the study cohort #1 (*n*=62) and #2 (*n*=86). Data from cohort 1 has been published previously [3]

Cohort #1		
	n / mean	%SD
Age (years)	63.98	± 6.06
pT stage	pT2a (*n* = 2)	3.2%
	pT2b (*n* = 2)	3.2%
	pT2c (*n* = 39)	62.9%
	pT3a (*n* = 11)	17.7%
	pT3b (*n* = 8)	12.9%
WHO/ISUP grade group	Group 2: 3 + 4 (*n* = 5)	8.1%
	Group 3: 4 + 3 (*n* = 23)	37.1%
	Group 4: 4 + 4 (*n* = 21)	33.9%
	Group 5: 4 + 5 (*n* = 13)	21%
Cohort #2		
	n / mean	%SD
Age (years)	63.43	± 6.74
pT stage	pT2a (*n* = 2)	2.3%
	pT2b (*n* = 1)	1.2%
	pT2c (*n* = 56)	65.1%
	pT3a (*n* = 23)	26.7%
	pT3b (*n* = 4)	4.7%
WHO/ISUP grade group	Group 1: 3 + 3 (*n*=5)	5.8%
	Group 2: 3 + 4 (*n* = 43)	50%
	Group 3: 4 + 3 (*n* = 25)	29.1%
	Group 4: 4 + 4 (*n* = 10)	11.6%
	Group 5: 4 + 5 (*n* = 3)	3.5%

#### Cohort #2

This cohort consisted of 86 RPE specimen. WHO/ISUP grade groups encompassed groups 1 to 5. Clinico-pathological characteristics are shown in Table 1.

### Immunohistochemistry

#### Cohort #1

**Fig. 2:**
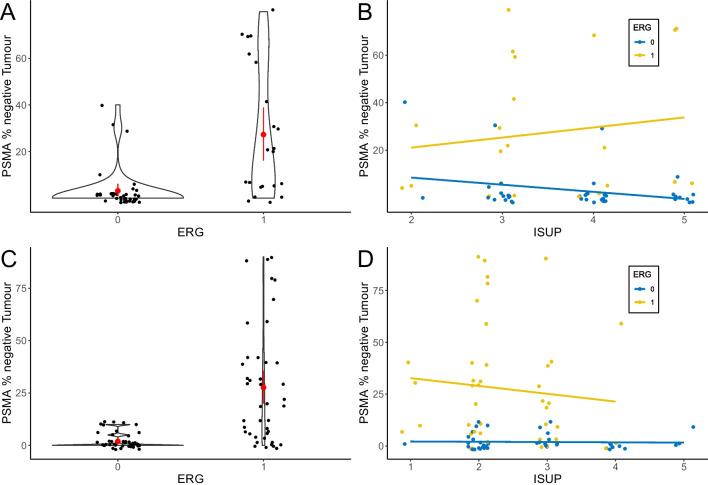
Correlation of IHC-PSMA negative tumour area and IHC-ERG expression in both cohorts. Cohort #1 (*n*=62) shows significantly higher levels of IHC-PSMA negative tumour areas in ERG positive cases (**A**), *p* < 0.001, Mann-Whitney U. No association of WHO/ISUP grade and IHC-PSMA expression, however cases with more than 40% PSMA_%neg_ showed all ERG reactivity (**B**). In the second cohort (*n*=86), similar results could be observed, with significantly lower levels of PSMA_%neg_ tumour area in ERG negative cases (*p*<0.001, Mann–Whitney U) depicted in (**C**). Correlation with WHO/ISUP grade groups showed a trend towards lower grade group in ERG positive cases, however this did not reach statistical significance (*p* = 0.072 Mann-Whitney U), whereas any case with at least 20% PSMA_%neg_ was ERG positive (**D**)

ERG expression was detected in 22 (35.5%) of 62 RPE specimens. PSMA expression was noted in all 62 (100%) adenocarcinoma specimens with a range from medium to strong membranous (2+ to 3+) and weak to strong (1+ to 3+) cytoplasmic expression. Intratumoural heterogeneous PSMA expression was observed in 38 of 62 cases (61.2%, Table 2). Twenty-five cases (40.3%) showed areas completely negative for PSMA comprising with a range from 20 to 95% of PSMA positive tumour area (PSMA_%pos_), corresponding to 5% to 80% PSMA_%neg_. Lesions without ERG expression had significantly less PSMA negative tumour areas, therefore a lower PSMA_%neg_ rate (mean 3.13% ± 9 vs. 27.27% ± 28.2 vs. as shown in Fig. 2A, with *p* < 0.001, Mann–Whitney U test). The correlation between ERG expression and WHO/ISUP grade group did not reach statistical significance (*p* = 0.143, Mann–Whitney U test, Fig. 2B).

**Table 2:** Different levels of PSMA heterogeneity stratified by IHC-ERG expression in both cohorts

	Homogeneous IHC-PSMA	Heterogeneous IHC-PSMA	Total
Cohort #1			
ERG negative	22	18	40
ERG positive	2	20	22
Total	24	38	62
Cohort #2			
ERG negative	18	26	44
ERG positive	2	40	42
Total	20	66	86
Cohort #1 +#2			
ERG negative	40	44	84
ERG positive	4	60	64
Total	44	104	148

#### Cohort #2

Evaluation of nuclear ERG expression revealed a positive staining in 42 out of 86 cases (48.8%). PSMA expression was noted in all adenocarcinoma specimen with a range from weak to strong membranous and cytoplasmic (1+ to 3+) expression.

Intratumoural heterogeneity of PSMA expression could be observed in 66 cases (76.7%, Table 2). 46 cases (53.5%) showed areas completely negative for PSMA comprising 5 to 90% of the tumour area (PSMA_%neg_). All ERG negative tumour areas (*n*=44) were mostly PSMA positive, with all being at least 90% IHC-PSMA positive, or a maximum of 10% PSMA_%neg_. ERG negative lesions however had a significantly lower average PSMA_%neg_ (mean 1.93% ± 3.6 vs. 27.62% ± 28.3; *p* < 0.001 Mann–Whitney U test, Fig. 2C)_._ ERG expression showed a trend to lower WHO/ISUP grades, however without statistical significance (*p* = 0.072, Mann-Whitney U test, Fig. 2D).

In both cohorts, lesions without ERG expression had significantly more often a homogenous, intense PSMA expression (Table 2; 55% and 41% respectively), while lesions with ERG expression were only rarely homogeneously PSMA positive (0.9%, and 4.8% respectively, *p* < 0.001, Fisher Exact test).

### Correlation of ERG and PSMA immunohistochemistry with SUV_max_ values Cohort #1

**Fig. 3:**
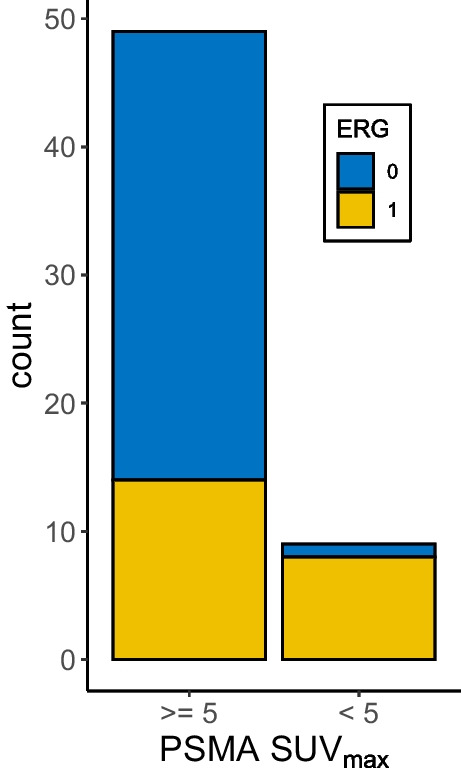
Distribution of IHC-ERG status in PSMA-PET positive and negative cases. Proportion of ERG positive cases (in yellow) in PSMA PET positive (SUV_max_ ≥ 5) and negative (SUV_max_ < 5) prostate cancers (staging cohort). Out of 49 PSMA-PET positive prostate cancers, 14 (29%) revealed a positive ERG expression while 35 (71%) were ERG negative. Out of 9 prostate cancers with negative PSMA PET, only one (11%) case was ERG negative, and 8 (89%) showed ERG expression.

Of the 62 patients in Cohort #1, 58 lesions were ≥ 5 mm, and considered for the following analysis. 49 patients had a clearly PSMA-positive PCa lesion in [^68^Ga]Ga-PSMA-11 PET, of which 35 (71%) were ERG negative, and only 14 (29%) had a positive ERG expression (Fig. 3). On the other hand, the proportion of ERG positive lesions in the group of PSMA negative PET was higher with 8 of 9 (88.9%), with only one lesion being ERG negative (Fig. 3).

**Fig. 4:**
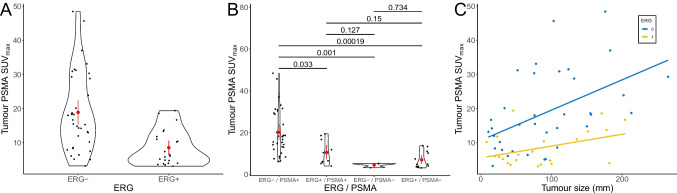
Correlation of IHC-ERG expression with SUV_max_ values in corresponding PSMA-PET scans and tumour sizes (cohort #1). (**A**) ERG positive PCa showed significantly lower SUV_max_ values in a corresponding PSMA PET scan (*p* < 0.01, Mann–Whitney U test). (**B**) Grouping into IHC-PSMA positive and negative cases (PSMA_%neg_ ≥ or < 20%), and including ERG status, significantly lower SUV_max_ values in IHC-PSMA positive cases are observed, when additionally expressing ERG (*p* = 0.033, Kruskal-Wallis test). No difference is seen in IHC-PSMA negative cases stratified by ERG expression (*p* = 0.734, Kruskal-Wallis test). In (**C**) ERG expression is depicted in association with SUV_max_ values and tumour size. It can be noted, that in tumours of similar size, ERG positive cases do show a lower SUV_max_ in the vast majority of cases

Additionally, lesions without IHC-ERG expression had significantly higher SUV_max_ values on corresponding [^68^Ga]Ga-PSMA-11-PET scans (mean SUV_max_ 18.88 ± 11.5 vs. 8.54 ± 5.16; *p* < 0.01, Mann–Whitney U test, Fig. 4A).

**Fig. 5:**
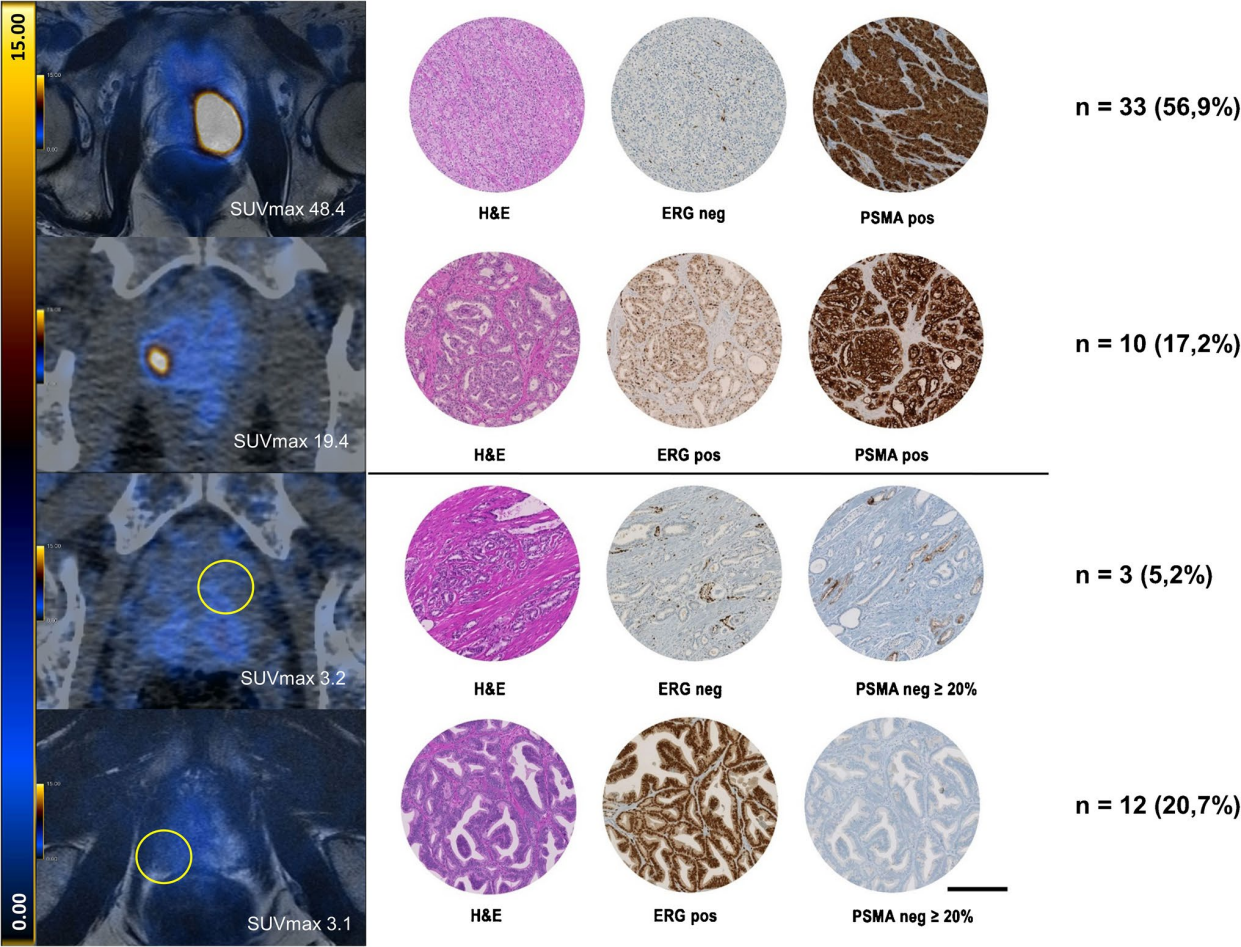
Distribution of IHC-ERG and IHC-PSMA positive and negative cases in correlation with PSMA PET imaging. The upper half shows cases with a positive IHC-PSMA expression (*n*=43, defined as PSMA_%neg_ in < 20% of the tumour area). Most of these cases (*n*=42, 97.7%) revealed a positive PET scan (examples on the left). In terms of ERG expression 33 (76.7%) cases had a negative and 10 (23.3%) a positive expression. The lower half displays the cases with a negative PSMA expression (*n*=15). Of those 8 (53%) had a negative PSMA PET scan (SUV_max_ < 5) and 12 (80%) had a positive ERG expression. Scale bar = 200 μm

In a subgroup analysis of IHC-PSMA positive prostate carcinomas (PSMA_%neg_ < 20%), an additional ERG positivity was significantly associated with lower SUV_max_ values in the corresponding PSMA PET scan (*p* = 0.033, Kruskal-Wallis test, Fig. 4B). However, in the subgroup of PSMA negative PCa (PSMA_%neg_ ≥ 20%), stratification of the ERG expression showed no significant differences in PSMA uptake on PET (*p*=0.734, Kruskal-Wallis test, Fig. 4B). The nine PET-negative cases were subjected to targeted RNA sequencing (Oncomine Focus Assay). The eight IHC-ERG positive cases showed corresponding *TMPRSS2::ERG* gene fusions, while the single IHC-ERG negative case did not reveal an ERG rearrangement (100% concordance between molecular analysis and IHC results). Evaluating the association of tumour size, IHC-ERG expression and SUV_max_ values on PET imaging, revealed no correlation between tumour size and IHC-ERG expression (*p* = 0.743, Mann Whitney U test). However, in the vast majority of cases with similar tumour sizes, IHC-ERG positive cases showed lower SUV_max_ (Figs. 4C and 5).

Combining both cohorts (*n*=148), analysis of WHO/ISUP grade group and ERG status showed statistical significance, with lower grade groups in the IHC-ERG positive group (*p* = 0.03, Mann-Whitney U test).

## Discussion

In this study, we show that PCa with IHC-ERG expression exhibit significantly more heterogeneous IHC-PSMA expression in two independent cohorts of primary PCa patients. This indicates a spatial regulatory effect, and increases the chance of PSMA negative areas, which are associated with negative PSMA PET scans, as shown in our previous study [3]. To estimate the overall IHC-PSMA expression on biopsies is difficult. Conversely, determining IHC-ERG status is easier to perform. The correlation between IHC-ERG positive carcinoma foci with significantly more and larger IHC-PSMA negative areas, and negative PSMA PET scans was confirmed in cohort #1. There were 35.5%, and 48.8% IHC-ERG positive cases, respectively in our two cohorts. This is in line with previous data ranging from 49% to 55% in the literature [16, 22]. The slightly lower fraction in cohort #1 might be explainable by a selection bias, as only intermediate to high-risk prostate adenocarcinoma have been included for primary staging with PSMA-PET imaging [3]. This is consistent with previous data that high PSMA expression in both PCa biopsies [23] and in *in vivo* PSMA-PET imaging [24], can serve as an independent prognostic factor for shorter recurrence free survival. Analysing the two cohorts independently from each other, there was no association between WHO/ISUP grade group and ERG expression; however when combining both cohorts, ERG positive cases showed clustering in significantly lower grade groups. IHC-ERG expression status did not reveal a correlation with tumour size. In similar tumour sizes, IHC-ERG expression was associated with a lower SUV_max_ in the vast majority of cases. IHC-ERG is strongly associated to an underlying *ERG* gene fusion, most often *TMPRSS2::ERG* [16, 17, 25]. We sequenced the 9 PSMA PET negative cases, of which 8 showed ERG expression. These 8 cases revealed a concomitant *TMPRSS2::ERG* gene fusion. The single PSMA-PET / IHC-ERG negative case did not reveal an ERG rearrangement. Looking at the IHC-ERG expression of IHC-PSMA positive cases (low PSMA_%neg_ fraction (< 20%)), the IHC-ERG positive cases showed more frequently lower SUV_max_ values. Interestingly, it has been shown in cell lines, that *TMPRSS2::ERG* gene fusion negatively regulates PSMA expression [11]. In particular, this mechanism seems to be associated with androgen receptor (AR) signalling, while increased AR signalling is correlated with enhanced expression of the *TMPRSS2::ERG* fusion transcript, and seems to interact with the PSMA enhancer (PSME) [11, 26]*.* However, as androgen receptor signalling is highly complex, and requires investigation from several perspectives, we cannot draw further conclusions from our data. Nevertheless our findings point to a negative regulation of PSMA expression in the IHC-ERG / *TMPRSS2::ERG* fusion positive subgroup of primary PCa. Conversely, IHC-ERG negative primary PCa have very few IHC-PSMA negative areas in the vast majority of cases. Thus, this group is a particularly attractive target for PSMA-based imaging and eventually even for focal therapy or theragnostic procedures.

Further studies are needed to substantiate these promising findings, with one advantage being the fast, easy, and reliable antibody staining of ERG on PCa tissue. Deeper investigation of the complex relationship between the negative regulation of PSMA by *ERG* fusion transcripts and other molecular pathways *in vivo* will help to better identify patients eligible for PSMA-based diagnostic and / or therapeutic approaches. In castration-resistant prostate carcinoma (CRPC) or advanced PCa, groups, distinct from our cohorts, PSMA regulation has been associated to HOXB13 expression, *FOLH1* (PSMA encoding gene) promotor methylation, and H3K27 methylation. In addition, the local microenvironment (“soil”) of the target organ of the metastasis seems to influence PSMA expression levels [9, 10]. Furthermore, DNA repair defects and DNA double strand breaks have been described to contribute to heterogeneity in PSMA expression levels [6, 7]. As mentioned, most of these studies have been performed in advanced and / or CRPC, which are usually heavily pre-treated and therefore might not represent the biology of the primary tumour [27]. In addition, IHC-ERG expression declines in a subgroup (20 - 26%) of *TMPRSS2::ERG* gene fusion positive PCa treated with androgen deprivation [28, 29]. Therefore, IHC-ERG might be a less reliable marker in the advanced / castration resistant clinical stage and the adverse effect on PSMA expression may decrease, although ERG reactivation has been documented [30]. In summary, currently available data corroborate a role of ERG expression in the negative regulation of PSMA expression, and should therefore be included in future research, in particular to assess its significance in the advanced clinical setting.

## Conclusion

This study describes immunohistochemical ERG expression as a parameter associated with a lower [^68^Ga]Ga-PSMA-11 uptake and less immunohistochemical PSMA expression in RPE specimen of primary PCa. Assessment of such parameters may serve as the basis of future biopsy-based biomarker development for an individualized imaging and therapy approach.

## Data Availability

The datasets generated during and/or analysed during the current study are available from the corresponding author on reasonable request.
